# Impact of head and neck cancer adaptive radiotherapy to spare the parotid glands and decrease the risk of xerostomia

**DOI:** 10.1186/s13014-014-0318-z

**Published:** 2015-01-09

**Authors:** Joel Castelli, Antoine Simon, Guillaume Louvel, Olivier Henry, Enrique Chajon, Mohamed Nassef, Pascal Haigron, Guillaume Cazoulat, Juan David Ospina, Franck Jegoux, Karen Benezery, Renaud de Crevoisier

**Affiliations:** Department of Radiotherapy, Centre Eugene Marquis, Avenue de la bataille Flandre Dunkerque, F-35000 Rennes, France; Rennes University 1, LTSI, Campus de Beaulieu, Rennes, F-35000 France; INSERM, U1099, Campus de Beaulieu, Rennes, F-35000 France; CHU Pontchaillou, Rennes, F-35000 France; Centre Antoine Lacassagne, Nice, F-06100 France

**Keywords:** Head and neck cancer, Anatomical variation, Adaptive RT, Xerostomia

## Abstract

**Background:**

Large anatomical variations occur during the course of intensity-modulated radiation therapy (IMRT) for locally advanced head and neck cancer (LAHNC). The risks are therefore a parotid glands (PG) overdose and a xerostomia increase.

The purposes of the study were to estimate:

- the PG overdose and the xerostomia risk increase during a “standard” IMRT (IMRT_std_);

- the benefits of an adaptive IMRT (ART) with weekly replanning to spare the PGs and limit the risk of xerostomia.

**Material and methods:**

Fifteen patients received radical IMRT (70 Gy) for LAHNC. Weekly CTs were used to estimate the dose distributions delivered during the treatment, corresponding either to the initial planning (IMRT_std_) or to weekly replanning (ART). PGs dose were recalculated at the fraction, from the weekly CTs. PG cumulated doses were then estimated using deformable image registration. The following PG doses were compared: pre-treatment planned dose, per-treatment IMRT_std_ and ART. The corresponding estimated risks of xerostomia were also compared. Correlations between anatomical markers and dose differences were searched.

**Results:**

Compared to the initial planning, a PG overdose was observed during IMRT_std_ for 59% of the PGs, with an average increase of 3.7 Gy (10.0 Gy maximum) for the mean dose, and of 8.2% (23.9% maximum) for the risk of xerostomia. Compared to the initial planning, weekly replanning reduced the PG mean dose for all the patients (p < 0.05). In the overirradiated PG group, weekly replanning reduced the mean dose by 5.1 Gy (12.2 Gy maximum) and the absolute risk of xerostomia by 11% (p < 0.01) (30% maximum). The PG overdose and the dosimetric benefit of replanning increased with the tumor shrinkage and the neck thickness reduction (p < 0.001).

**Conclusion:**

During the course of LAHNC IMRT, around 60% of the PGs are overdosed of 4 Gy. Weekly replanning decreased the PG mean dose by 5 Gy, and therefore by 11% the xerostomia risk.

## Introduction

**Figure 1:**
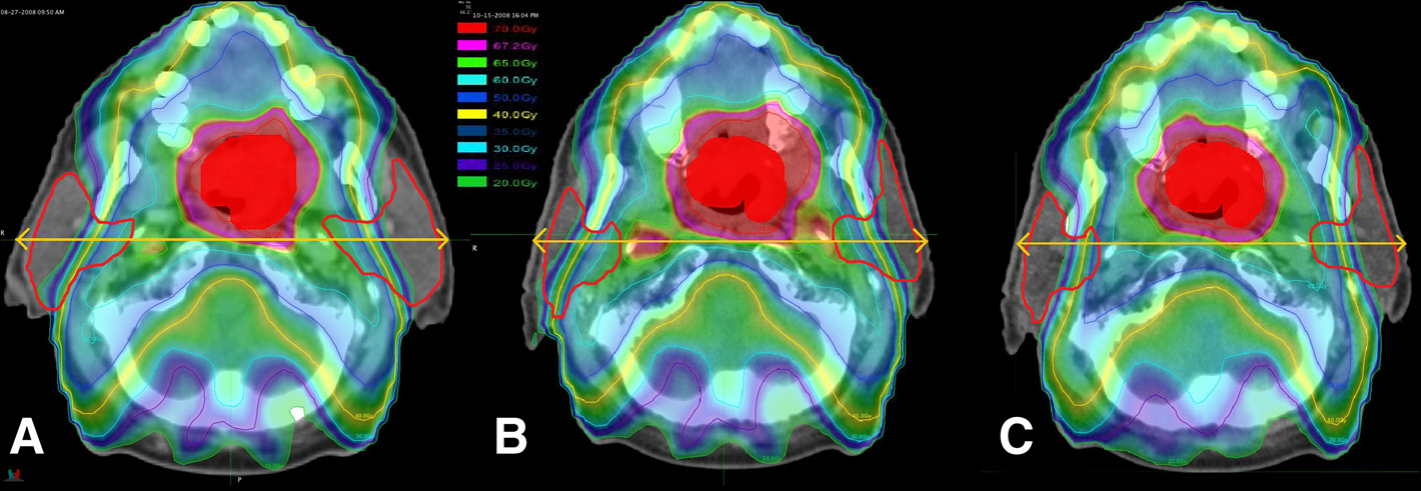
**Illustration of the anatomical variations on the dose distribution.** IMRT dose distributions at different times for a given patient, showing the PG overdose without replanning **(B)** and the benefit of replanning **(C). A**: Planned dose on the pre-treatment CT (CT0). **B**: Actual delivered dose without replanning during the treatment (Week 3). **C**: Adaptive planned dose with replanning to spare the parotid glands (PG) at the same fraction (Week 3). PGs are shown by the red line. The full red represents the Clinical Target Volume (CTV70). The arrow show the head thickness. Figure 1B and 1C compared to 1A shows that the PGs and the CTV70 volumes and the neck thickness have decreased. These anatomical variations have led to dose hotspots in the neck, close to the internal part of the two PG (Figure 1B). Replanning (Figure 1C) allowed to spare the PG even better than on the planning (Figure 1A).

The treatment of unresectable Head & Neck Cancer (HNC) consists of a chemoradiotherapy [1,2]. One of the most common toxicity of this treatment is xerostomia, inducing difficulties in swallowing and speaking, loss of taste, and dental caries, with therefore a direct impact on patient quality of life. Xerostomia is mainly caused by radiation induced damage mainly to the parotid glands (PG), and to a lesser extend to the submandibular glands [3]. Intensity modulated radiotherapy (IMRT) permits to deliver highly conformal dose in complex anatomical structures, while sparing critical structures. Indeed, three randomized studies have demonstrated improving (PG) sparing by using IMRT compared to non-IMRT techniques, resulting in better salivary flow and decreased xerostomia risk [4-6]. However, large variations can be observed during the course of IMRT treatment, such as body weight loss [7,8], primary tumor shrinking [7], and PG volume reduction [9]. Due to these anatomical variations and to the tight IMRT dose gradient, the actual administered dose may therefore not correspond to the planned dose, with a risk of radiation overdose to the PGs (Figure 1) [10,11]. This dose difference clearly reduces the expected clinical benefits of IMRT, increasing the risk of xerostomia. Although bone-based image-guided radiation therapy (IGRT) allows for setup error correction, the actual delivered dose to the PGs remains higher than the planned dose [12], due to the fact that IGRT does not take shape/volume variations into account. By performing one or more new planning during the radiotherapy treatment, adaptive radiotherapy (ART) aims to correct such uncertainties. ART has been already shown to decrease the mean PG dose during locally advanced head and neck cancer IMRT [13], but no surrogate of the PG dose difference and of the dosimetric benefit of ART has yet been identified. In the context of IMRT for locally advanced HNC, this study sought to:

- estimate the difference between the planned dose and the actual delivered dose (without replanning) to the PGs, i.e., the PG overdose;
- estimate the PG dose difference with replanning and without replanning to spare the PGs while keeping the same planning target volume (PTV), i.e., the benefit of ART;
- identify anatomical markers correlated with these dose differences (PG overdose and ART benefit).

## Materials and methods

### Patients and tumors

The study enrolled a total of 15 patients with a mean age of 65 years (ranging from 50 to 87 years). Patient, tumor, and treatment characteristics are provided on Table 1. All tumors were locally advanced (Stage III or IV, AJCC 7th ed). The mean PG volume was 25.3 cc (ranging from 16.6 cc to 52.1 cc, standard deviation (SD): 8.1 cc).

**Table 1:** **Patient, tumor, and treatment characteristics at the initial planning (CT0)**

**ID**	**Gender**	**Age**	**Tumor localization**	**TNM**	**Volume (cc)**			**D**_**mean**_ **(Gy)**		**Xerostomia NTCP (%)** **[** 21 **]**	
					**CTV70**	**HLP**	**CLP**	**HLP**	**CLP**		
										**HLP**	**CLP**
1	M	86	Tonsil	T3N1	45.2	52.1	48.6	30.2	31.1	26.5	28.3
2	F	63	Tonsil	T2Nx	26.3	31.1	27.5	31.4	26	29.0	18.7
3	M	74	Oropharynx	T3N2c	181.5	24.9	20.7	37.9	31.1	44.3	28.4
4	F	66	Oropharynx	T2N2c	107.2	27.8	23.4	32.9	27.9	32.3	22.0
5	M	57	Velum	T3N0	62.4	20.7	18.0	28.1	27.8	22.4	21.7
6	M	67	Oropharynx	T3N2c	156.2	24.5	22.7	30.8	29.4	24.7	21.4
7	M	52	Oropharynx	T4N2	165.1	N/A	21.6	N/A	28.7	N/A	23.4
8	M	67	Trigone	T4N1	139.3	22.0	19.3	30.7	29.2	27.4	24.4
9	F	65	Oropharynx	T3N3	237.5	23.9	20.2	42.4	31.1	55.2	28.2
10	F	65	Oropharynx	T4N3	257.9	N/A	24.5	N/A	35.2	N/A	37.7
11	M	50	Oropharynx	T4N2c	434.5	N/A	17.7	N/A	36.3	N/A	40.3
12	M	53	Oropharynx	T3N0	14.4	16.6	23.3	41.3	24.2	52.9	15.9
13	M	73	Oropharynx	T3N2c	147.0	29.4	29.2	54.6	32.2	81.7	30.7
14	M	56	Larynx	T3N0	14.0	22.8	29.2	19.7	9.2	10.1	2.7
15	M	75	Hypopharynx	T2N2	76.3	20.3	22.4	29.4	29.1	25.0	24.4

M: male; F: female; CT0: initial planning; CTV70: clinical target volume receiving 70 Gy; PGs: parotid glands; HLP: homolateral PGs; CLP: contralateral PGs; Dmean: mean dose at initial planning; N/A: not applicable (PGs included in the CTV), NTCP: normal tissue complication risk of xerostomia defined as a salivary flow ratio <25% of the pretreatment one [21].

### Treatment and planning

All patients underwent IMRT using a total dose of 70 Gy (2 Gy/fraction/day, 35 fractions), with a simultaneous integrated boost technique [14] and concomitant chemotherapy. Planning CTs (CT0) with intravenous contrast agents were acquired with 2 mm slice thickness from the vertex to the carina. A thermoplastic head and shoulder mask with five fixation points was used. PET-CT and MRI co-registration was used for tumor delineation. Three target volumes were generated. Gross tumor volume (GTV) corresponded to the primary tumor along with involved lymph nodes. Clinical target volume 70 Gy (CTV_70_) was equal to GTV plus a 5 mm 3D margin, which was adjusted to exclude air cavities and bone mass without evidence of tumor invasion. CTV_63_ corresponded to the area at high-risk of microscopic spread, while CTV_56_ corresponded to the prophylactic irradiation area. GTV, CTV_63_, CTV_56,_ and all organs at risk were manually delineated on each CT slice. Adding a 5 mm 3D margin around the CTVs generated the PTVs. PTV expansion was limited to 3 mm from the skin surface in order to avoid the build-up region and to limit skin toxicity [15]. All IMRT plans were generated using Pinnacle V9.2. Seven Coplanar 6-MV photon beams were employed with a step and shoot IMRT technique. The prescribed dose was 70 Gy to PTV_70_, 63 Gy to PTV_63,_ and 56 Gy to PTV_56_. The collapsed cone convolution/superposition algorithm was used for dose calculation. The maximum dose within the PTV was 110% (D2%). The minimum PTV volume covered by the 95% isodose line was 95%. Dose constraints were set according to the GORTEC recommendations [16]: a mean dose (D_mean_) <30 Gy and a median dose <26 Gy for contralateral PGs.

Patients were treated as planned on CT0 and no changes were applied to dose distribution during treatment. During the treatment course, weekly in-room stereoscopic imaging corrected set-up errors >5 mm. All patients signed an informed consent form. The study was approved by the institutional review board (ARTIX study NCT01874587).

### Weekly dose estimations, in cases of replanning and without replanning

During the treatment, each patient underwent six weekly CTs (CT1 to CT6) according to the same modalities as CT0, except for the intravenous contrast agents (not systematically used, particularly in case of cisplatin based chemotherapy). For each patient, the anatomical structures were manually segmented on each weekly CT by the same radiation oncologist. In case of complete response, initial macroscopically-involved areas were still included in the CTV_70_, which was adjusted to exclude any air cavities and bone mass without evidence of initial tumor invasion.

**Figure 2:**
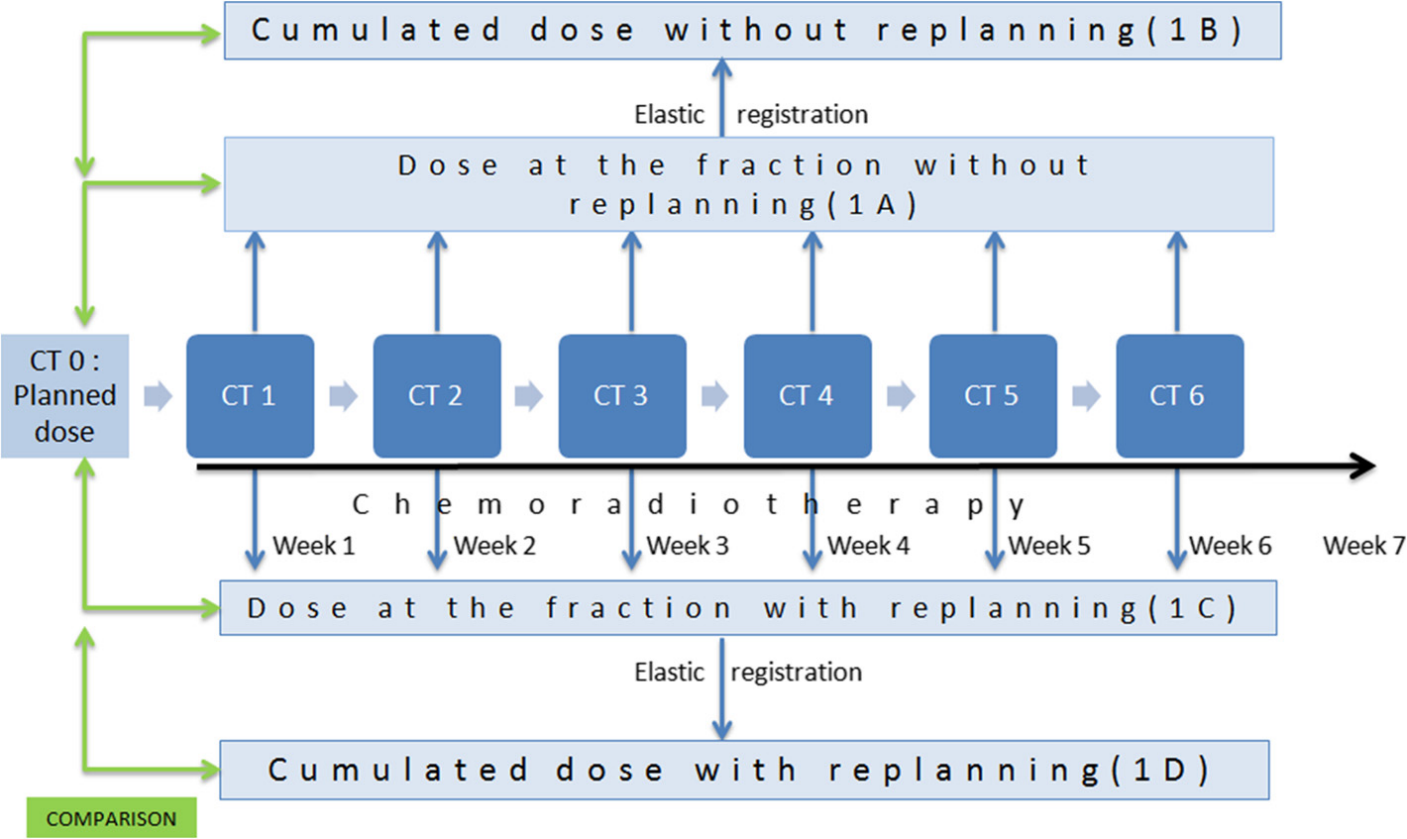
**Overall study flow chart.** Weekly CT scans were performed during the 7 weeks of treatment. Doses were calculated on each weekly fraction, corresponding either to the initial planning (step 1A) or to a replanning to spare the parotid glands (step 1C). Corresponding cumulated doses were calculated (steps 1B and 1C) using elastic registration. Doses were then compared.

Actual weekly doses (Figure 2, Step 1A) were estimated by calculating the dose distribution on the weekly CT, using treatment parameters and isocenter from CT0. Weekly re-planned doses (Figure 2, Step 1C) were calculated by generating a new IMRT plan on each weekly CT in accordance with the dose constraints described for the initial planning. PTV coverage did not differ between initial planning and weekly re-planned CT. The dose constraints for the organs at risk have respected the GORTEC recommendations at the initial planning and in all replanning.

### Total cumulated dose estimations by deformable registration

Cumulated doses were estimated for the two scenarios, with or without replanning (Figure 2 Steps 1B and 1D), according to the following deformable image registration procedure (contour-guided Demons registration algorithm) [17]. For PGs on each CT, a signed distance map was generated to represent the squared Euclidean distance between each voxel and the PG surface. Distance maps of each PG were then registered using the Demons registration algorithm [18]. The resulting deformation fields were employed to map the weekly dose distributions to the planning CT using tri-linear interpolation. Next, the mapped dose distributions were summed to estimate the cumulated dose for each PG. The average Dice score for PG registration, from the weekly CT to each planning CT was computed as followed:

Dice score = 2×(|A∩B|)/(|A| + |B|), where: A is the delineated PG contour on the weekly CT, B is the planning contour propagated by the registration and |.| denotes the number of voxels encompassed by the contour. The Dice score ranges from 0 (worst case: no match between both contours) to 1 (perfect match) [19]. A 3D dose difference in the PG was calculated between the cumulated dose distribution and planned dose distribution.

### Anatomical variation description

Anatomical variations (between CT0 and weekly CTs) were characterized by variations in CTV_70_ and PG volumes, in the distances between PGs and CTV_70_ and in the thickness of the neck (at the level of the geometrical centers of the PGs). The distance between PGs and CTV_70_ corresponded to the minimal distance between the surfaces of the two contours (PG-CTVds), computed using an Euclidean distance map of the first contour, iteratively considering all the points of the second contour and keeping the resulting minimal distance.

### Statistical analysis

The impact of the anatomical variations on PG dose was analyzed considering D_mean_ and the full DVH. Their impact on the risk of xerostomia was estimated by using the LKB NTCP model (n = 1, m = 0.4, and TD50 = 39.9) [20,21], the complication being defined as a salivary flow ratio <25% of the pretreatment one [22].

The mean PG dose differences between the weekly doses (with and without replanning) and the planned dose were calculated (Figure 2). The PG overdose was assessed as the difference between the dose without replanning (at the fraction or cumulated) and the dose at the planning. The benefit of weekly replanning was assessed as the difference between the doses with replanning and without replanning (at the fraction or cumulated). Linear mixed-effects models were used to test if the following parameters were correlated with the PG overdose or the benefit of the weekly replanning: initial volumes of the CTV_70_ and of the PGs, decreasing (between the weekly CT and the planning CT) of the volume of the CTV_70_ (in cc and %) and the PGs (in cc and %), shortening of the distance between PGs and CTV_70_, reduction of the head thickness and the time between the CT0 and the beginning of treatment. All dose and volume comparisons were performed using nonparametric tests (Wilcoxon test). Statistical analysis was carried out using the Statistical Package for the Social Sciences V 20.0.

## Results

Since 3 ipsilateral PGs were completely included within the PTV (Patient number 7, 10 and 11), they were excluded from the analysis, resulting in a total of 27 PGs analyzed. The average Dice score [19] for PG registration, from the weekly CT to each planning CT was 0.92 (from 0.83 to 0.95).

### Quantification of anatomical variations during the 7 weeks of treatment

From CT0 to CT6, the PG volumes decreased by a mean value of 28.3% (ranging from 0.0 to 63.4%, SD 18%), corresponding to an average decrease of 1.1 cc/week (ranging from 0.0 to 2.2 cc/week). The CTV_70_ decreased by a mean value of 31% (ranging from 73% to −13%, SD 28%).

The distance between the PGs and the CTV (PG-CTV_ds_) decreased in 74% of the PGs by 4.3 mm on average (ranging from 0.1 to 12 mm, SD 3.7 mm), whereas it increased in the other 26% of the PGs by 3.2 mm on average (ranging from 1.1 to 6.3 mm, SD 2.1 mm).

The thickness of the neck decreased for 78% of the patients by a mean value of 7.9 mm (ranging from 0.1 to 26.6 mm, SD 6.2 mm).

### Dose comparison between planned dose, and doses with or without replanning in PGs

The per-treatment PG doses (with or without replanning) were analyzed, first considering the weekly fractions and then, using the cumulated doses from all weekly fractions, for all the 15 patients. The results are shown in Table 2.

**Table 2:** **Parotid gland overdose and replanning benefit assessments, based on the fraction or the cumulated doses, for all the 15 patients**

	**D**_**mean**_ **(Gy),mean (Min-max;SD)**		**p-value**
	Planned dose (1)	30.9 (9.2-54.6; 7.9)	-
Doses at the fraction	Without replanning (2)	33.0 (7.7-61.2; 9.9)	
	With replanning (3)	29.4 (4.1-51.7; 8.3)	
	PG overdose (4) = (2)-(1)	1.8 (−10.6-24.9; 5.8)	<0,001
	Replanning benefit (5) = (3)-(2)	3.8 (0–23.8; 4.0)	<0,001
Cumulated doses	Without replanning (2)	32.0 (8.7-57.6; 9.3)	-
	With replanning (3)	28.6 (4.6-51.2; 8.4)	
	PG Overdose (4) = (2)-(1)	1.1 (−7.9-10.0; 4.1)	0,1
	Replanning benefit (5) = (3)-(2)	3.6 (0–12.2; 3.3)	<0,001

PGs: parotid glands; Dmean: First, the mean PG dose was calculated for each patient and each week (DmeanWeekly). Then, the mean of the DMeanWeekly was calculated for each patient (DMeanPt). Finally, the mean of the DmeanPt was calculated for the whole population (D(mean)).

p values are calculated using the Wilcoxon test, to test if the Dmean in (1) and (2), and if the Dmean in (2) and (3) are statistically different.

#### PG dose comparisons at the per-treatment weekly fraction

**Figure 3:**
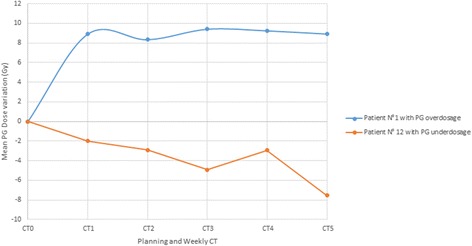
**Variation over time of the mean PG dose for two representative patients.** Red line corresponding to patient N°1 who presenting an increasing of the mean PG dose cumulated. Blue line corresponding to the patient N°12 who presenting a decreasing of the mean PG dose cumulated.

In order to assess the PG overdose, comparison was first made between the dose at the fraction without replanning (Figure 2 Step 1A) and the planned dose. For 67% of the plans, the D_mean_ increased on average by 4.8 Gy (up to 24.9 Gy, SD 4.6 Gy). In the other 33% of plans, the D_mean_ decreased by 3.9 Gy (up to 10.7 Gy, SD 2.9 Gy). The variation of the mean PG dose during the treatment was showed Figure 3 for two representative patients.

Then, to assess the benefit of replanning, comparison was made between the dose with (Figure 2 step 1C) and without replanning (Figure 2 Step 1A). In 85% of the plans, replanning decreased the D_mean_ on average by 4.6 Gy (up to 23.8 Gy, SD 4.0 Gy).

#### PG dose comparisons using the cumulated doses and the corresponding estimated xerostomia risks

**Figure 4:**
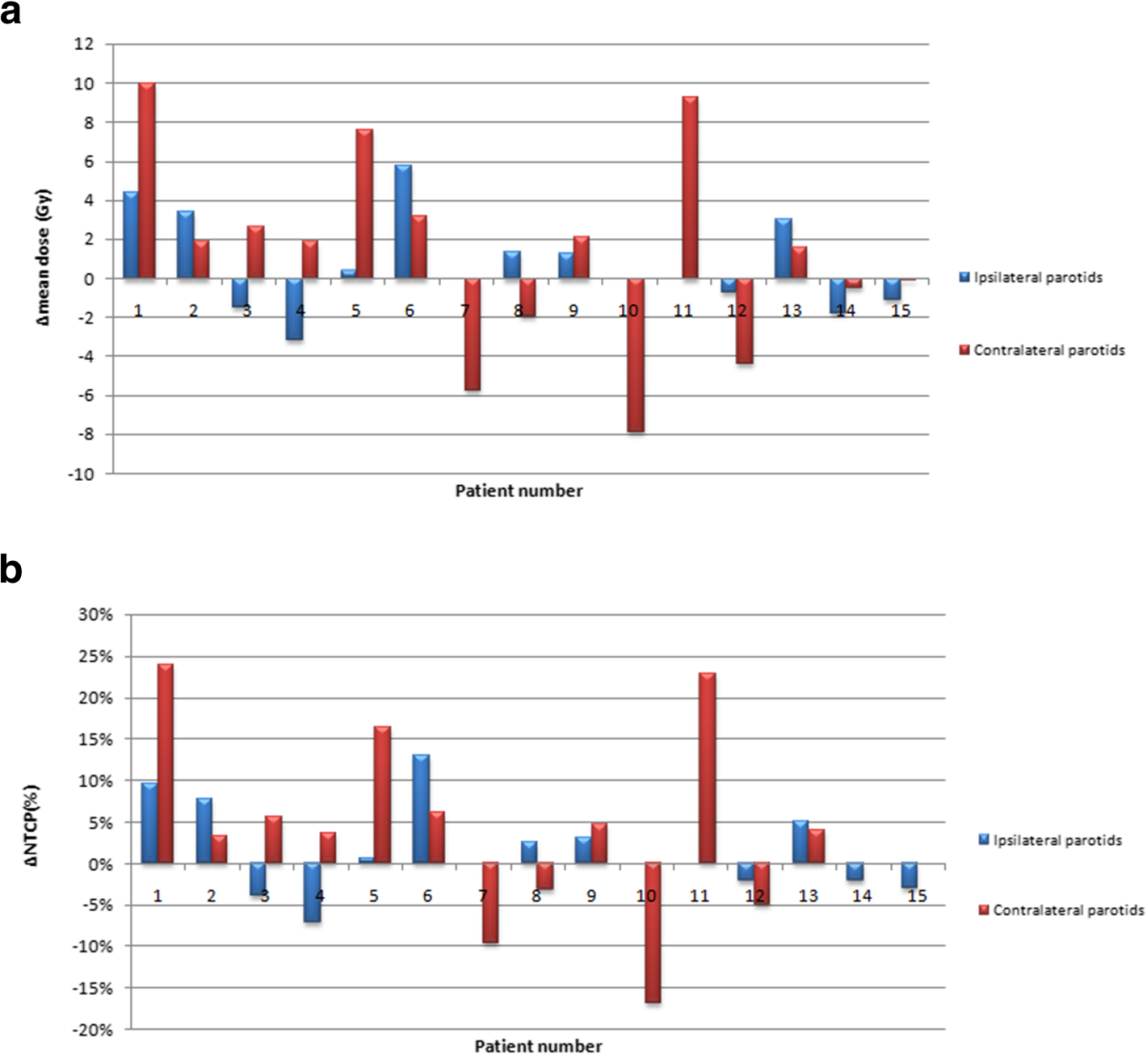
**Parotid gland overdose assessment: Difference between the mean cumulated dose (without replanning) and the mean dose at the planning, in each of the parotid gland, for each of the 15 patients (** **4** **a).** The corresponding impact on the xerostomia risk (%) is presented Figure **4** **b.** NTCP: normal tissue complication risk of xerostomia defined as a salivary flow ratio <25% of the pretreatment one [21].

**Figure 5:**
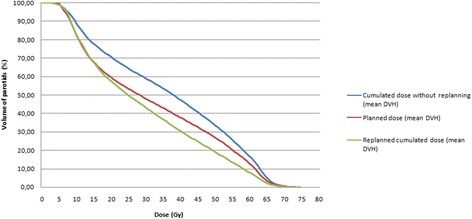
**Mean parotid gland dose-volume histograms (DVHs) showing the impact of replanning on the over-irradiated PGs (n = 16).**

A PG overdose was reported in 59% (N = 16) of the PGs. Figure 4a shows the D_mean_ difference for each PG of each patients. Ten out of fifteen patients received a higher D_mean_ in at least one PG (6 patients in the 2 PGs), which corresponded to a D_mean_ increase of an average of 3.7 Gy (ranging from 0.4 to 10.0 Gy, SD 2.9 Gy). Figure 5 shows the average planned DVH (red line) and the average cumulated DVH without replanning (blue line).

Figure 4b shows the corresponding difference in the estimated xerostomia risk. The average absolute increased risk of xerostomia was 3% (ranging from −16.7 to 23.9%, SD 2.9%) in all patients, and was 8.2% (ranging from 3.8 to 23.9%, SD 7.1%) among patients with an increased dose to PGs.

**Figure 6:**
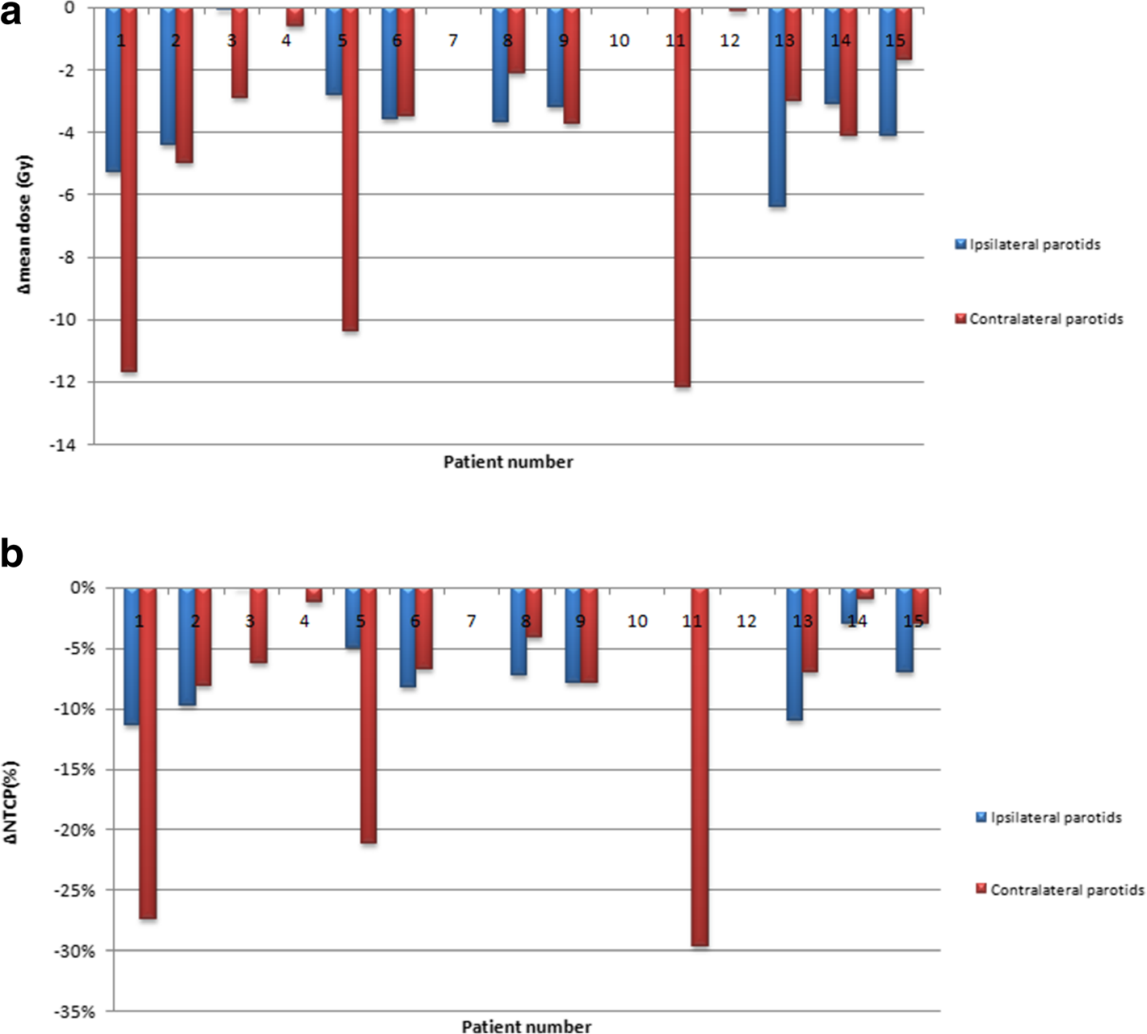
**Replanning benefit assessement: cumulated mean dose difference between the dose with replanning and the dose without replanning, in each of the parotid gland (ipsilateral and contralateral), for each of the 15 patients (6a), and corresponding estimated xerostomia risk (%) (6b).** NTCP: normal tissue complication risk of xerostomia defined as a salivary flow ratio <25% of the pretreatment one [21].

Weekly replanning enabled the D_mean_ to be reduced to at least the same value as that of the pre-treatment planning for all over-irradiated PGs (Figure 6). In the subgroup of over-irradiated PGs, the mean D_mean_ difference between the cumulated doses with replanning and without replanning was therefore 5.1 Gy (ranging from 0.6 to 12.2 Gy, SD 3.3 Gy) (p = 0.001). In the subgroup of non-over-irradiated PGs, this mean D_mean_ difference was 1.4 Gy (ranging from 0 to 4.1 Gy, SD 1.7 Gy) (p = 0.001). Figure 5 displays the impact of the replanning to decrease the PG dose, with the average cumulated DVH with replanning (green line) and without replanning (blue line).

In the over-irradiated PG group, the replanning decreased the xerostomia risk by 11% on average (ranging from 1 to 30%, SD 8%) (p < 0.01).

### Anatomical parameters correlated with PG overdose or replanning benefit

PG overdose and replanning benefit (at the fraction or cumulated) increased with the CTV_70_ shrinkage and the reduction of neck thickness (p < 0.01). At the fraction, a reduction of 10 cc of the CTV_70_ or of 1 mm of the neck thickness leads to an increase of the mean PG dose of 0.3 Gy. The PG volume variation has no impact on the mean PG dose.

## Discussion and conclusion

The main goal of definitive chemoradiotherapy in locally advanced HNC is to improve locoregional control, while keeping a high quality of life. Reducing the dose in the PGs during the whole course of IMRT and therefore xerostomia is a major challenge. Indeed, we found the majority of the PGs (59%) being overirradiated of a mean dose of 4 Gy (up to 10 Gy), resulting to an absolute increase risk of xerostomia of 8% (up to 24%). The ART strategy appears to benefit not only to the over-irradiated PG patients, reducing the mean dose of 5 Gy (up to 12 Gy) and the xerostomia risk of 11% (up to 30%), but also to the non-over-irradiated PGs. These results suggest thus a large use of ART for the majority of locally advanced HNC patients. In our study, four patients (N° 4, 7, 10 and 12) have not clear benefit from replanning. These patients were presented a spontaneous decrease of the mean PG dose during the treatment. No more gain was possible with the replanning due to the other constraints (homogeneity, spinal cord, brainstem …). The GORTEC dose volume constraints has been respected for all the replanning.

The dosimetric benefit of ART has been shown in a limited number of studies, and not exclusively for the PGs. In a series of 22 patients, Schwartz *et al*. evaluated the impact of one and two replanning using daily CT on rails [23]. The mean PG dose was decreased of 3.8% for contralateral PGs and of 9% for ipsilateral PGs, with possible sparing of the oral cavity and larynx. In another series of 20 patients, a single replanning performed at the 3rd or 4th week of treatment decreased the mean PG dose of 10 Gy [9]. On the other hand, Castadot *et al*. didn’t show any dosimetric benefits for PGs when using four replanning in a series of 10 patients, however reducing the spinal cord dose and improving the CTV_56_ dose conformation [24].

The optimal number and time of replanning are unclear. Wu *et al*. concluded that one replanning decreased the mean PG dose by 3%, two replanning by 5%, and six replanning by 6% [13]. A “maximalist” weekly replanning strategy was considered feasible in our study, as in an ongoing randomized study (ARTIX) comparing one IMRT based planning to a weekly based IMRT replanning. The benefit of such strategy has to be demonstrated compared to other replanning strategies. Ongoing study (like ARTFORCE trial) test the benefit of only one replanning [24]. The benefit of each supplementary weekly replanning has to be evaluated. A true adaptive RT strategy should be personalized to each patient, ranging potentially from no re-planning to a maximalist weekly replanning. Ideally, replanning decisions may likely be based on either geometrical criteria or cumulated dose monitoring corresponding to the dose-guided RT approach. Replanning is also particularly time-consuming, complete delineation taking up to 2.5 hours in our experience and that of others [25-28]. Deformable image co-registration software can be used to propagate the OAR contours from the initial planning CT to the per-therapeutic planning CT, reducing the delineation time by approximately a factor 3 [26,28]. The CTV delineation should be however carefully checked, to prevent recurrence due to inadequately reduced CTV. Indeed, the goal of ART in our study was to spare the PGs during treatment as they were spared at the planning, while keeping the same appropriate CTV coverage (and not to reduce the CTV coverage).

The analysis of the anatomical variations occurring within the course of IMRT is crucial to understand the overdose of the PGs and to identify early the sub-group of the overdosed PGs (59%). We found that mean volumes decreased by 28% for the PGs and 31% for the CTV, in agreement with the literature reporting values of 15% to 28% for the PGs, 69% for the GTV, and 8% to 51% for the CTV [7,9,13,23,26,29]. We found that the PGs overdose (without replanning) and the dosimetric benefit of replanning increased with the tumor shrinkage and the reduction of head thickness. The last one is likely explained by loss of weight, tumor shrinking and decrease of the PG volume. The reduction of the head thickness leads consequently to the occurrence of dose hotspot in the neck, close or within the PGs (Figure 1). Other studies also found that reduction of the neck diameter increases the risk of over-irradiation [30,31]. The variation of the mean PG dose was more important between the CT0 and the CT1 than between each weekly CT. This difference may be explained by the delay between CT0 and the first weekly CT. In our study, the PG dose differences between the fraction and the initial planning are likely related to both the set-up error (we did not quantified) and the anatomical structures volume/shape variations. Systematic set-up errors may increase the mean PG dose by around 3% by mm of displacement [32]. This point suggests, for a daily practice, to combine both a daily bone registration to correct the set-up errors and replanning to correct the anatomical variations.

Fraction comparison only provides information for a specific moment and there is a need for full treatment dose evaluation and comparison. Deformable registration enables dose fraction accumulation [33]. Since PG shape and volume variations were limited, our study’s Dice scores were relatively high (0.92). However, the Dice score does not provide any information regarding the registration’s anatomical “point to point” correspondence accuracy. Moreover, the possibility of PG defects observed over the course of radiotherapy [34] should prompt careful consideration of this cumulated dose approach, thereby justifying an independent “fraction to fraction” dose analysis. Our results, based on both weekly fraction and cumulated dose, were consistent. The 3D dose visualization and differential DVH of the dose difference between the cumulated dose and planning dose (Figure 1) revealed moreover the heterogeneity of hotspot distribution in PGs, which may also impact on the xerostomia risk. The cranial part of the PGs seems to be more critical [35,36], maybe due to the presence of an important concentration of salivary gland stem cells at this level [37]. The possible heterogeneity of the radiosensitivity within the PG could be therefore more carefully investigated in order to consider to spare not only the full gland (represented by a mean dose endpoint) but also subparts of the gland. Indeed, relatively small dose (10 Gy) within the PG may cause severe loss of function [38], and dose greater than 20 Gy may cause up to 90% loss of the acinar cells [39]. It seems also that radiation-induced gland dysfunction are due to membrane damage, causing secondarily necrosis of acinar cells and atrophy of the lobules [40].

Our study exhibits limitations. The small patient number did not allow us to analyze the potential impact of tumor localization. Even if CTs from a single patient were always delineated by the same radiation oncologist, intra-observer variabilities in organ delineation are also potentially responsible for uncertainties. Moreover, the clinical benefit of the weekly replanning has been estimated and was not reported in the study.

In conclusion, an ART strategy combining a daily bone registration and a weekly replanning may be proposed for locally advanced HNC, with an expected benefit to decrease xerostomia. This PG-sparing strategy appears however particularly complex and should be therefore assessed within prospective trials, with a special attention for CTV delineation. The optimal number and time of replanning are unclear. The benefit of a weekly replanning strategy versus other replanning strategies have to been demonstrated.

## References

[1] St Guily JL, Borget I, Vainchtock A, Remy V, Takizawa C. Head and neck cancers in France: an analysis of the hospital medical information system (PMSI) database. Head Neck Oncol. 2010;2:22. 10.1186/1758-3284-2-22 PMC2942880 20809978

[2] Pignon JP, Bourhis J, Domenge C, Designe L (2000). Chemotherapy added to locoregional treatment for head and neck squamous-cell carcinoma: three meta-analyses of updated individual data. MACH-NC Collaborative Group. Meta-Anal Chemother Head Neck Cancer Lancet.

[3] Chambers MS, Rosenthal DI, Weber RS (2007). Radiation-induced xerostomia. Head Neck.

[4] Kam MK, Leung SF, Zee B, Chau RM, Suen JJ, Mo F (2007). Prospective randomized study of intensity-modulated radiotherapy on salivary gland function in early-stage nasopharyngeal carcinoma patients. J Clin Oncol.

[5] Nutting CM, Morden JP, Harrington KJ, Urbano TG, Bhide SA, Clark C (2011). Parotid-sparing intensity modulated versus conventional radiotherapy in head and neck cancer (PARSPORT): a phase 3 multicentre randomised controlled trial. Lancet Oncol.

[6] Pow EH, Kwong DL, McMillan AS, Wong MC, Sham JS, Leung LH (2006). Xerostomia and quality of life after intensity-modulated radiotherapy vs. conventional radiotherapy for early-stage nasopharyngeal carcinoma: initial report on a randomized controlled clinical trial. Int J Radiat Oncol Biol Phys.

[7] Barker JL, Garden AS, Ang KK, O’Daniel JC, Wang H, Court LE (2004). Quantification of volumetric and geometric changes occurring during fractionated radiotherapy for head-and-neck cancer using an integrated CT/linear accelerator system. Int J Radiat Oncol Biol Phys.

[8] Duma MN, Kampfer S, Schuster T, Winkler C, Geinitz H (2012). Adaptive radiotherapy for soft tissue changes during helical tomotherapy for head and neck cancer. Strahlenther Onkol.

[9] Nishi T, Nishimura Y, Shibata T, Tamura M, Nishigaito N, Okumura M (2013). Volume and dosimetric changes and initial clinical experience of a two-step adaptive intensity modulated radiation therapy (IMRT) scheme for head and neck cancer. Radiother Oncol: J Eur Soc Ther Radiol Oncol.

[10] Hansen EK, Bucci MK, Quivey JM, Weinberg V, Xia P (2006). Repeat CT imaging and replanning during the course of IMRT for head-and-neck cancer. Int J Radiat Oncol Biol Phys.

[11] Lee C, Langen KM, Lu W, Haimerl J, Schnarr E, Ruchala KJ (2008). Assessment of parotid gland dose changes during head and neck cancer radiotherapy using daily megavoltage computed tomography and deformable image registration. Int J Radiat Oncol Biol Phys.

[12] O’Daniel JC, Garden AS, Schwartz DL, Wang H, Ang KK, Ahamad A (2007). Parotid gland dose in intensity-modulated radiotherapy for head and neck cancer: is what you plan what you get?. Int J Radiat Oncol Biol Phys.

[13] Wu Q, Chi Y, Chen PY, Krauss DJ, Yan D, Martinez A (2009). Adaptive replanning strategies accounting for shrinkage in head and neck IMRT. Int J Radiat Oncol Biol Phys.

[14] Kutcher GJ, Burman C (1989). Calculation of complication probability factors for non-uniform normal tissue irradiation: the effective volume method. Int J Radiat Oncol Biol Phys.

[15] Mohan R, Wu Q, Manning M, Schmidt-Ullrich R (2000). Radiobiological considerations in the design of fractionation strategies for intensity-modulated radiation therapy of head and neck cancers. Int J Radiat Oncol Biol Phys.

[16] Lee N, Chuang C, Quivey JM, Phillips TL, Akazawa P, Verhey LJ (2002). Skin toxicity due to intensity-modulated radiotherapy for head-and-neck carcinoma. Int J Radiat Oncol Biol Phys.

[17] Gérard JP, Ortholan C, Pointreau Y (2010). Normal tissue tolerance to external beam radiation therapy. Cancer Radiother.

[18] Cazoulat G, Simon A, Dumenil A, Gnep K, de Crevoisier R, Acosta O (2014). Surface-constrained nonrigid registration for dose monitoring in prostate cancer radiotherapy. IEEE Trans Med Imaging.

[19] Thirion JP (1998). Image matching as a diffusion process: an analogy with Maxwell’s demons. Med Image Anal.

[20] Lyman JT (1985). Complication probability as assessed from dose-volume histograms. Radiat Res Suppl.

[21] Dijkema T, Raaijmakers CP, Ten Haken RK, Roesink JM, Braam PM, Houweling AC (2010). Parotid gland function after radiotherapy: the combined michigan and utrecht experience. Int J Radiat Oncol Biol Phys.

[22] Dice LR (1945). Measures of the amount of ecologic association between species. Ecology.

[23] Schwartz DL, Garden AS, Shah SJ, Chronowski G, Sejpal S, Rosenthal DI (2013). Adaptive radiotherapy for head and neck cancer–dosimetric results from a prospective clinical trial. Radiother Oncol: J Eur Soc Ther Radiol Oncology.

[24] Castadot P, Geets X, Lee JA, Gregoire V (2011). Adaptive functional image-guided IMRT in pharyngo-laryngeal squamous cell carcinoma: is the gain in dose distribution worth the effort?. Radiother Oncol: J Eur Soc Ther Radiol Oncol.

[25] Berwouts D, Olteanu LA, Duprez F, Vercauteren T, De Gersem W, De Neve W et al.: Three-phase adaptive dose-painting-by-numbers for head-and-neck cancer: initial results of the phase I clinical trial. Radiotherapy and oncology: journal of the European Society for Therapeutic Radiology and Oncology 2013. 10.1016/j.radonc.2013.04.002 23647760

[26] Budach W, Bolke E, Fietkau R, Buchali A, Wendt TG, Popp W et al.: Evaluation of time, attendance of medical staff, and resources during radiotherapy for head and neck cancer patients: the DEGRO-QUIRO trial. Strahlenther Onkol, 187:449–460. 10.1007/s00066-011-2273-z 21786109

[27] Daisne JF, Blumhofer A. Atlas-based automatic segmentation of head and neck organs at risk and nodal target volumes: a clinical validation. Radiat Oncol. 2013;8:154. 10.1186/1748-717X-8-154 PMC3722083 23803232

[28] Nishimura Y, Nakamatsu K, Shibata T, Kanamori S, Koike R, Okumura M (2005). Importance of the initial volume of parotid glands in xerostomia for patients with head and neck cancers treated with IMRT. Jpn J Clin Oncol.

[29] Lai YL, Yang SN, Liang JA, Wang YC, Yu CY, Su CH, et al. Impact of body-mass factors on setup displacement in patients with head and neck cancer treated with radiotherapy using daily on-line image guidance. Radiat Oncol. 2014;9:19. 10.1186/1748-717X-9-19 PMC3904466 24411006

[30] You SH, Kim SY, Lee CG, Keum KC, Kim JH, Lee IJ (2012). Is there a clinical benefit to adaptive planning during tomotherapy in patients with head and neck cancer at risk for xerostomia?. Am J Clin Oncol.

[31] Delana A, Menegotti L, Bolner A, Tomio L, Valentini A, Lohr F (2009). Impact of residual setup error on parotid gland dose in intensity-modulated radiation therapy with or without planning organ-at-risk margin. Strahlenther Onkol.

[32] Castadot P, Lee JA, Parraga A, Geets X, Macq B, Gregoire V (2008). Comparison of 12 deformable registration strategies in adaptive radiation therapy for the treatment of head and neck tumors. Radiother Oncol: J Eur Soc Ther Radiol Oncol.

[33] Fiorino C, Rizzo G, Scalco E, Broggi S, Belli ML, Dell’Oca I (2012). Density variation of parotid glands during IMRT for head-neck cancer: correlation with treatment and anatomical parameters. Radiother Oncol: J Eur Soc Ther Radiol Oncol.

[34] Konings AWT, Cotteleer F, Faber H, van Luijk P, Meertens H, Coppes RP (2005). Volume effects and region-dependent radiosensitivity of the parotid gland. Int J Radiat Oncol Biol Phys.

[35] Konings AWT, Faber H, Cotteleer F, Vissink A, Coppes RP (2006). Secondary radiation damage as the main cause for unexpected volume effects: A histopathologic study of the parotid gland. Int J Radiat Oncol Biol Phys.

[36] Lombaert IM, Brunsting JF, Wierenga PK, Faber H, Stokman MA, Kok T, et al. Rescue of salivary gland function after stem cell transplantation in irradiated glands. PLoS One. 2008;3:e2063. 10.1371/journal.pone.0002063 PMC2329592 18446241

[37] Bussels B, Maes A, Flamen P, Lambin P, Erven K, Hermans R (2004). Dose–response relationships within the parotid gland after radiotherapy for head and neck cancer. Radiother Oncol.

[38] Henriksson R, Frojd O, Gustafsson H, Johansson S, Yi-Qing C, Franzen L (1994). Increase in mast cells and hyaluronic acid correlates to radiation-induced damage and loss of serous acinar cells in salivary glands: the parotid and submandibular glands differ in radiation sensitivity. Br J Cancer.

[39] Porter SR, Fedele S, Habbab KM (2010). Xerostomia in head and neck malignancy. Oral Oncol.

[40] Heukelom J, Hamming O, Bartelink H, Hoebers F, Giralt J, Herlestam T, et al. Adaptive and innovative Radiation Treatment FOR improving Cancer treatment outcomE (ARTFORCE); a randomized controlled phase II trial for individualized treatment of head and neck cancer. BMC Cancer. 2013;13:84. 10.1186/1471-2407-13-84 PMC3599345 23433435

